# Ribavirin Exerts Differential Effects on Functions of Cd4^+^ Th1, Th2, and Regulatory T Cell Clones in Hepatitis C

**DOI:** 10.1371/journal.pone.0042094

**Published:** 2012-07-27

**Authors:** Bettina Langhans, Hans Dieter Nischalke, Simone Arndt, Ingrid Braunschweiger, Jacob Nattermann, Tilman Sauerbruch, Ulrich Spengler

**Affiliations:** Department of Internal Medicine I, University of Bonn, Bonn, Germany; University of Sydney, Australia

## Abstract

Ribavirin improves outcomes of therapy in chronic hepatitis C but its mode of action has still remained unclear. Since ribavirin has been proposed to modulate the host’s T cell responses, we studied its direct effects on CD4^+^ T cell clones with diverse functional polarization which had been generated from patients with chronic hepatitis C. We analysed in vitro proliferation ([^3^H] thymidine uptake) and cytokine responses (IL-10, IFN-gamma) at varying concentrations of ribavirin (0–10µg/ml) in 8, 9 and 7 CD4^+^ TH1, TH2 and regulatory T cell (Treg) clones, respectively. In co-culture experiments, we further determined effects of ribarivin on inhibition of TH1 and TH2 effector cells by Treg clones. All clones had been generated from peripheral blood of patients with chronic hepatitis C in the presence of HCV core protein. Ribavirin enhanced proliferation of T effector cells and increased production of IFN-gamma in TH1 clones, but had only little effect on IL-10 secretion in TH2 clones. However, ribavirin markedly inhibited IL-10 release in Treg clones in a dose dependent fashion. These Treg clones suppressed proliferation of T effector clones by their IL-10 secretion, and in co-culture assays ribavirin reversed Treg-mediated suppression of T effector cells. Our *in vitro* data suggest that - in addition to its immunostimulatory effects on TH1 cells - ribavirin can inhibit functions of HCV-specific Tregs and thus reverses Treg-mediated suppression of T effector cells in chronic hepatitis C.

## Introduction

Hepatitis C virus (HCV) infection is a major gobal cause of liver cirrhosis and liver cancer [1], which is treated with interferon-based combination therapies. Adding the nucleoside analogue ribavirin to pegylated interferon (PEG-IFN) markedly improves outcomes of antiviral therapy [2]. Nevertheless its mode of action has remained elusive. At present, four hypotheses have been proposed comprising 1) inhibition of the host enzyme IMPDH, 2) inhibition of the NS5B-encoded RNA-dependent RNA polymerase (RdRp), 3) action of ribavirin as a putative RNA mutagene, and 4) improved HCV clearance by the immune system due to a ribavirin-induced switch of T cell polarization towards the more antiviral TH1 profile [3].

Although data have remained controversial, resolution of HCV infection appears to be linked to strong HCV-specific T cell responses [4]. In particular, HCV elimination under PEG-IFN/ribavirin combination therapy seems to be associated with improved HCV-specific T helper (TH) cell responses, which, however, peak either late during treatment or after achieving a therapeutic response [5]–[6]. Several lines of evidence indicate that the host antiviral response may be compromised during chronic HCV infection: high serum levels of IL-4 and IL-10 have been observed in patients with chronic hepatitis C [7]–[8], and a favourable clinical response to interferon therapy appeared to be associated with a reduction of TH2 cytokine levels and promotion of TH1-mediated immune responses [9]–[10].

Ribavirin is a nucleoside analogue, which reduces hepatic inflammation but exerts only little effect on HCV RNA levels [11]. An immunodulatory role of ribavirin has been deduced from studies in mice and from in vitro stimulation experiments with human CD4^+^ and CD8^+^ T cells using either mitogen or superantigen for stimulation [12]–[14]. Whereas these studies suggested simultaneous down-regulation of TH2 cytokines and up-regulation of TH1 cytokines, a recent in vitro stimulation study of PBMC with HCV antigens confirmed IL10 down-regulation but failed to detect up-regulated production of IFN-gamma [15]. Likewise, ex vivo studies in patients under treatment did not uniformly confirm up-regulation of interferon-gamma [5], [9]. In summary, the putative immunomodulatory functions of ribavirin have remained controversial but reduced production of IL-10 seems to be a key difference between responders and non-responders to IFN/ribavirin combination therapy.

HCV-specific T effector cells appear to be impaired by a variety of diffent mechanisms also comprising activation of CD4+ regulatory T cells (Tregs) [16]–[18]. Tregs express Foxp3 (forkhead transcription factor box3), produce IL-10, and can suppress cytokine-production and proliferation of virus-specific T effector cells in a contact-dependent manner [16]–[18]. Thus, Tregs may contribute importantly to HCV persistence and resistance to immune-based antiviral therapy. Recently we succeeded in establishing HCV core-specific CD25+CD4+ T cells clones from patients with chronic hepatitis C, which to some extent seem to reflect functions and phenotype of HCV-specific adaptive CD4+ Tregs [19].

To clarify the effect of ribavirin on functions of Tregs we compared its direct in vitro action on our adaptive Treg clones to that of autologous TH1 and TH2 polarized T effector cell clones obtained from the same patient with chronic hepatitis C.

## Materials and Methods

### Ethics Statement

The reported studies were approved by the Institutional Review Boards of the Bonn University Ethics Committee. Written informed consent was obtained from the patients prior to sample collection. Samples were coded and data stored anonymously.

### Study Groups

T cell clones were established from the peripheral blood of treatment-naïve, non-cirrhotic patients with chronic hepatitis C genotype 1 (8 males and 4 females; age: 45 (30–68) years, median (range)).

### Reagents

Recombinant HCV core protein (genotype 1, amino acids (aa) 1–115; <4.0 pg/µg LPS) was purchased from Mikrogen GmbH (Martinsried, Germany). Human recombinant IL-2 (rIL-2) and OKT-3 (human anti-CD3) were obtained from R&D Systems (Wiesbaden, Germany), anti-CD28 from BD Biosciences (Heidelberg, Germany) and cytomegalovirus (CMV) glycoprotein p65 from AUSTRAL Biologicals (San Ramon, California, USA).

OpTmizer™ T-Cell Expansion SFM medium and Dynabeads^®^ Human Treg Expander (Invitrogen, Karlsruhe, Germany) were used for cell culture. Synthetic ribavirin was purchased from Sigma-Aldrich Chemie GmbH (Taufkirchen, Germany).

### Generation of HCV-specific T Cell Clones

Peripheral blood mononuclear cells (PBMC) were isolated by Ficoll-Paque gradient centrifugation (PAA Laboratories GmbH, Linz, Austria) from heparinized blood of patients with chronic hepatitis C. Freshly isolated PBMC (5x10^5^cells/ml) were stimulated with recombinant HCV core protein (10 µg/ml) in the presence of rIL-2 (100 U/ml). After 10 days, cells were re-stimulated with core protein and supplemented with irradiated autologous feeder cells (5x10^5^ PBMC/ml). After 20 days, cells were seeded in Terasaki plates (Greiner bio-one, Frickenhausen, Germany) under conditions of limiting dilution (0.1, 0.3, and 0.5 cells/well). After 10 days, clones were picked from plates with less than 10% positive wells and re-stimulated at 10–14 day intervals in the presence of IL-2 and Treg Expander beads. Clones were classified into TH1, TH2 cells and Tregs by analyzing cytokine secretion and proliferation after stimulation with anti-CD3/anti-CD28 (1 and 2.5 µg/ml) (table 1). Overall we obtained 8, 9, and 7 TH1, TH2 and Treg clones from the 12 HCV-infected patients of this study, respectively. TH1 cells were IFN-gamma^high^, IL-4^low^ and IL-10^negative^. TH2 cells were IFN-gamma^negative^, IL-4^high^ and produced variable amounts of IL-10. The antigen-specificity of TH1 and TH2 clones was undetermined. Tregs were IFN-gamma^negative^, IL-4^negative^, IL-10^positive^ and did not proliferate after stimulation with anti-CD3/anti-CD28. All Treg clones expressed a typical Treg phenotype (CD25^+^, Foxp3^+^, CD127^−^, CTLA-4^+^) and in addition showed HLA-DR-restricted specificity for HCV. Of note, they could be induced to release IL-10 in response to recombinant HCV core - but not to a CMV control protein - as described in great detail elsewhere [19].

**Table 1:** Cytokine profiles and proliferation of Treg, TH2 and TH1 clones from patients with chronic hepatitis C.

	IL-4(pg/ml)	IL-10(pg/ml)	IFN-gamma(pg/ml)	poliferation(SI)
**Treg clones**				
#1	7	178	1	0
#2	29	730	5	1
#3	25	96	5	1
#4	15	69	1	1
#5	1	957	1	1
#6	33	194	27	2
#7	0	474	1	2
**TH2 clones**				
#8	671	519	4	21
#9	719	81	10	23
#10	1514	157	1	15
#11	661	219	6	10
#12	999	352	10	13
#13	901	254	8	19
#14	1283	615	20	21
#15	642	286	1	15
#16	640	54	0	31
**TH1 clones**				
#17	40	3	9648	35
#18	36	1	5085	15
#19	16	21	2512	42
#20	13	2	8010	36
#21	6	0	4100	7
#22	0	0	2523	15
#23	6	3	1206	15
#24	20	23	893	19

Based on the stimulation of control cells, cytokine values equal or greater 50 pg/ml were considered positive.

### Effect of Ribavirin on Functions and Phenotype of T Cell Clones

We stimulated TH1, TH2 and Treg clones with anti-CD3/anti-CD28 in the absence or presence of ribavirin various concentrations of (0, 1, 5 and 10 µg/ml) and measured their proliferative responses and cytokine production. Proliferation was studied via [^3^H] thymidine up-take assays on day 5, as described previously [19]. Stimulation indices (SI) were calculated as the ratio of [^3^H] thymidine uptake relative to the medium controls. SIs>4 were considered positive.

72 h after stimulation anti-CD3/anti-CD28 IFN-gamma was measured in the supernatants of TH1 clones, and IL-10 was measured in the supernatants of TH2 and Treg clones. IL-10 and IFN-gamma were captured with primary antibodies JES3-9D7 and NIB42, and bound cytokines detected with the corresponding biotin-labeled antibodies JES3-268 and S.B3 (all BD Biosciences), respectively. Cytokine/antibody complexes were revealed by horseradish peroxidise-conjugated streptavidin using TMB (tetramethylbenzidin**e**) as a substrate. Plates were read in an ELISA reader (Tecan, Crailsheim, Germany) at 450 nm.

**Figure 1:**
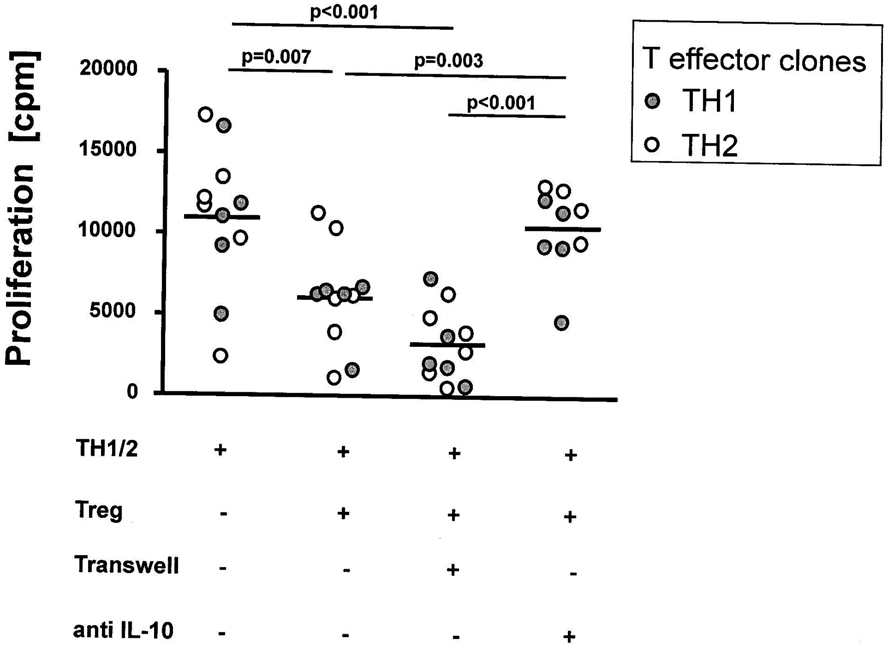
Inhibition of TH cell proliferation by CD4^+^ Treg clones. This figure illustrates that Treg clones generated from patients with chronic hepatitis C inhibit proliferation of autologous anti-CD3/anti-CD28 stimulated TH1 and TH2 clones. Inhibition of TH1 and TH2 clones was not altered when direct cell-contact was prevented by co-culture in a semipermeable transwell system. Co-culture experiments with a neutralising IL-10 antibody (10 µg/ml; clone 23738) prevented Treg-mediated suppression of TH1 and TH2 clones indicating that inhibition of TH1 and TH2 clones by Treg clones involved IL-10 production as a major soluble inhibitor. Proliferative responses were studied via [^3^H] thymidine assays. The figures present mean±SEM of experiments with TH1 (filled circles) and TH2 clones (open circles), respectively.

Using flow cytometry (CANTO II, BD Bioscience) and antibodies APC anti-human CD152 (clone L3D10) and FITC anti-human CD39 (clone A1) (both Biolegend, Germany) we analysed expression in Treg clones at ribavirin concentrations of 0, 1, 5, 10 µg/ml.

To study potential effects of ribavirin on the expansion phase of Tregs we studied if ribavirin (1, 5, 10 µg/ml) affected the proliferation of anti-CD3/anti-CD28 stimulated Tregs in the presence of high amounts of recombinant IL-2 (100 IU/ml).

Ribavirin is frequently used in combination with IFN-alpha, which also regulates the dynamic balance in T cell subsets [20]. Typical concentrations under treatment of hepatitis C are 1–5 µg/ml for ribavirin [21], [22] and 10–100 IU/ml for IFN-alpha, respectively [21], [23]. Therefore, we analysed the effects of ribavirin on IL-10 secretion in Tregs in the presence of 100 IU/ml Interferon alfa-2a (Roferon-A, Roche; kindly provided by Dr. S. Zahn, Department of Dermatology, University of Bonn).

**Figure 2:**
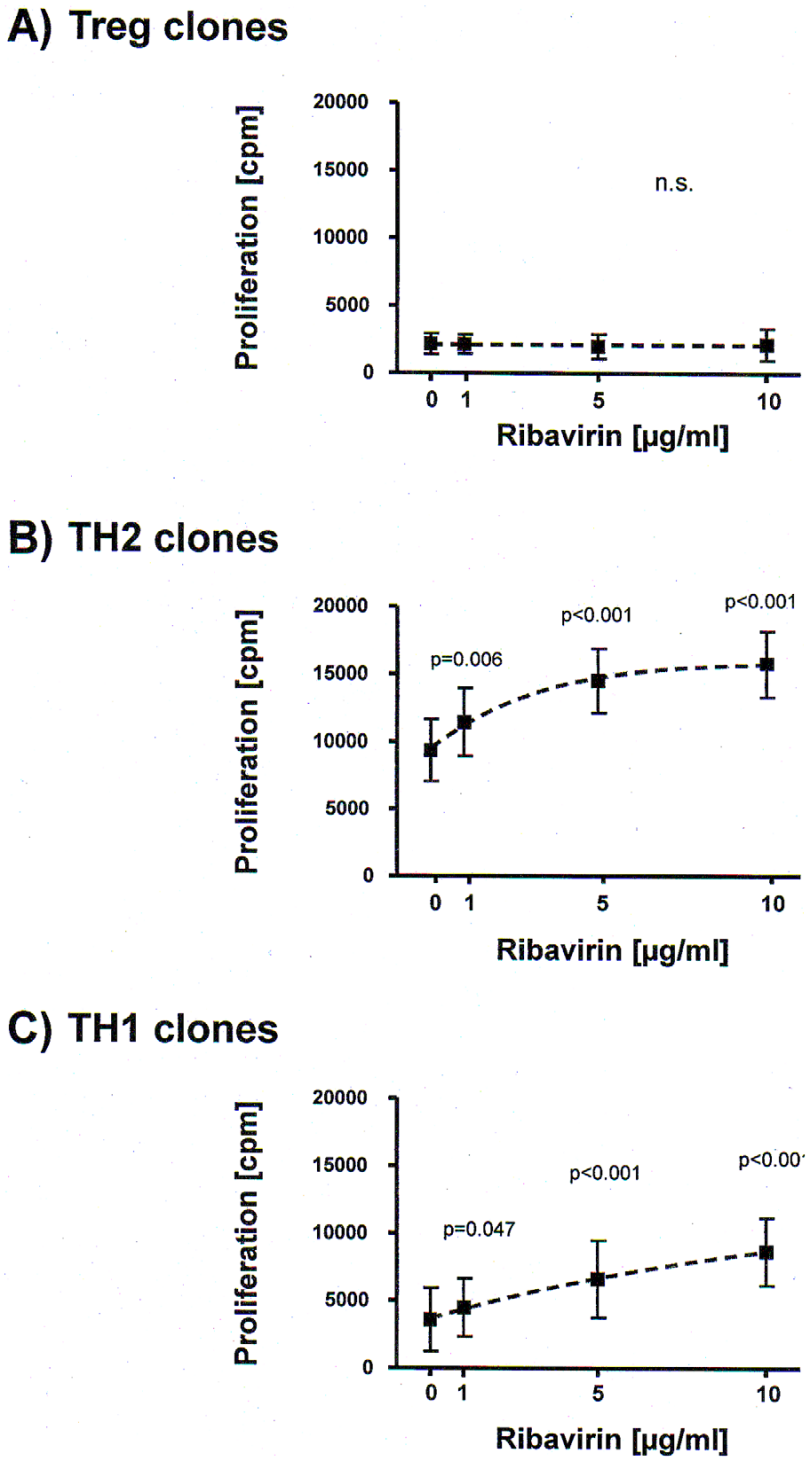
Effect of ribavirin on proliferation of differentially polarized CD4^+^ T cell clones. Figure 2 shows dose response curves of proliferation ([^3^H] thymidine uptake, cpm = counts per minute; mean ± SEM) versus ribavirin concentrations (0, 1, 5 and 10 µg/ml) in anti-CD3/anti-CD28-activated Treg clones (A; n = 7), TH2 clones (B; n = 8) and TH1 clones (C; n = 7). Response curves were obtained by a non-linear curve fit with the GraphPad Prism Response Curve module (GraphPad Prism 4 Software, San Diego California, USA). Statistical significance was calculated by 1-way ANOVA with correction for repeated measurements.

**Figure 3:**
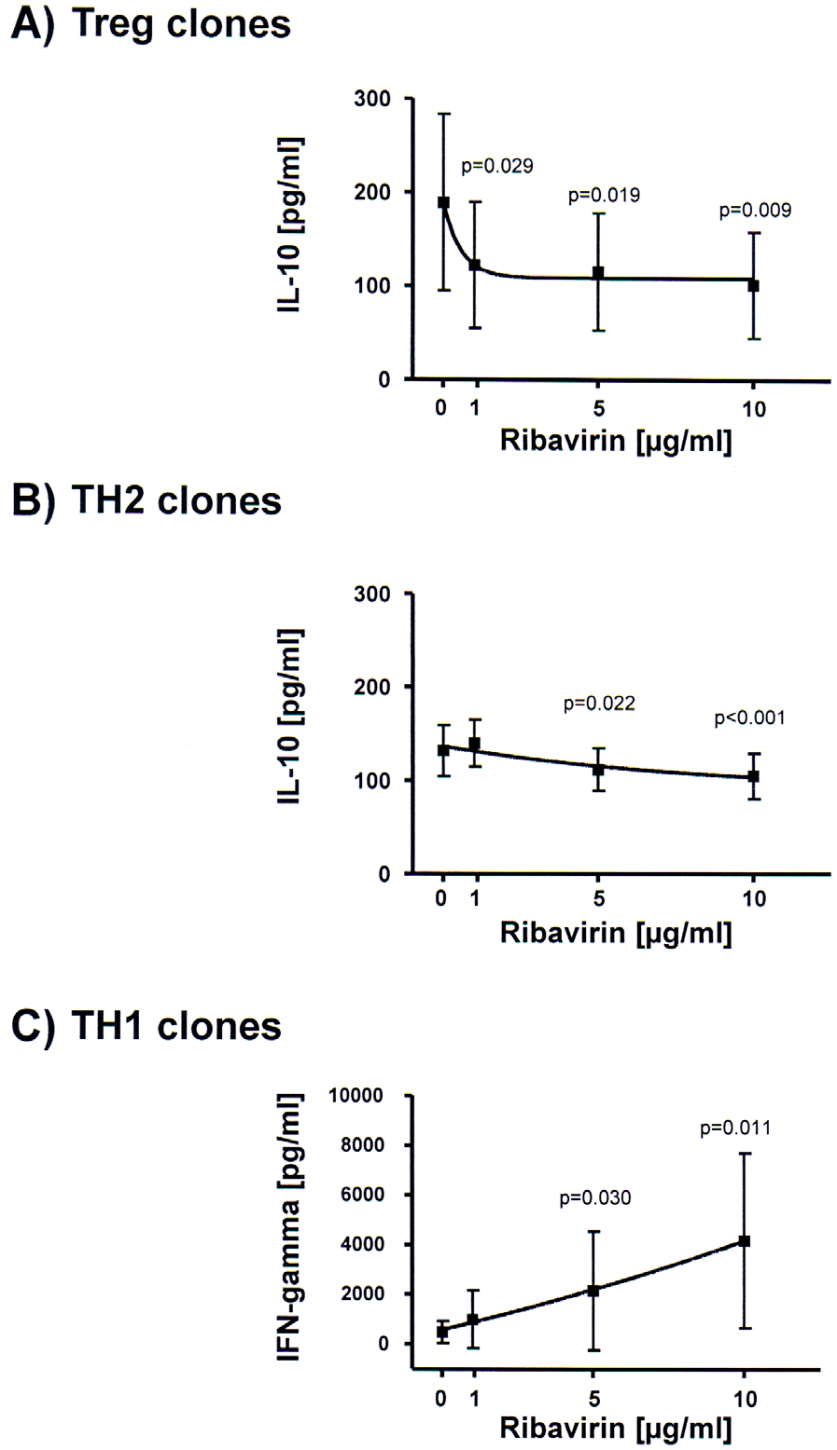
Effect of ribavirin on cytokine secretion in differentially polarized CD4^+^ T cell clones. Figure 3 illustrates dose response curves of cytokine release (pg/ml) versus ribavirin concentrations (0, 1, 5 and 10 µg/ml) in anti-CD3/anti-CD28-activated Treg clones (A; n = 7), TH2 clones (B; n = 9) and TH1 clones (C; n = 8). Dose response curves were calculated analogous to figure 2.

### Influence of Ribavirin on mRNA Levels of Transcription Factors in T Cell Clones

To check if ribavirin alters mRNA expression levels of transcription factors T-bet and GATA-3 in TH1 and TH2 clones we also measured changes of T-bet and GATA-3 upon stimulation with ribavirin by quantitative real time PCR on a Light Cycler® instrument (Roche, Germany).

First, total RNA was extracted using the RNeasy Mini Kit (Qiagen, Germany) according to the manufacturer’s instructions. Elimination of genomic DNA and reverse transcription was carried out using the QuantiTect Reverse Transcription Kit (Qiagen, Germany) according to the manufacturer’s standard protocol.

For each PCR run 1 µL of the obtained cDNA was used as template. PCR was carried out in a final volume of 10 µL on a LightCycler instrument using the LightCycler FastStart DNA-Master SYBR Green 1 kit (Roche Molecular Diagnostics, Mannheim, Germany). Primers of the house-keeping gene ß-actin were purchased from TibMolbiol (Berlin, Germany): sense 5′-TGGCATCGTGATGGACTCC-3′, antisense 5′ AATGTCACGCACGATTTCCC-3′. Analysis of T-bet and GATA-3 mRNA expression was conducted with commercially available QuantiTect Primer Assays following the instructions of the manufacturer (T-bet: Hs_TBX21_1_SG, GATA-3: Hs_GATA3_1_SG; Qiagen). The amplification protocol consisted of an initial denaturation step at 95°C for 10 min, followed by 40 cycles (denaturation at 95°C for 2 sec, annealing at 60°C for 5 sec, extension at 72°C for 15 sec) and fluorescence acquisition at 72°C. PCR products were identified by melting curve analysis (95°C for 10 sec, 65°C for 15 sec and a slow ramp (0.2°C/sec) to 95°C with continuous fluorescent acquisition). The LightCycler® software version 3.5 was used in all PCR experiments.

**Figure 4:**
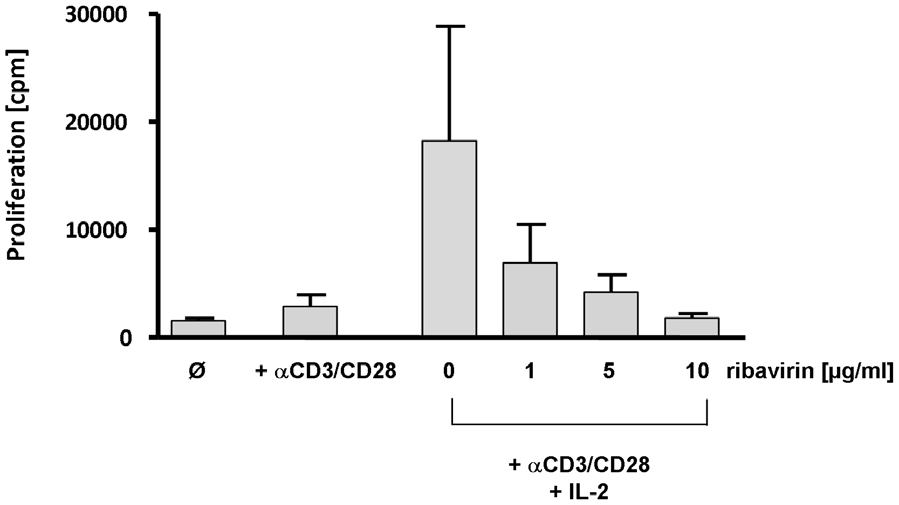
Influence of ribavirin on the proliferation of Treg clones in the presence of IL-2. Figure 4 demonstrates that anti-CD3/anti-CD28-stimulated Tregs markedly proliferate in the presence of high amounts of recombinant IL-2 (100 IU/ml). Adding ribavirin at various concentrations (0, 1, 5 10 µg/ml) proliferation of Tregs was significantly reduced under these experimental conditions.

**Figure 5:**
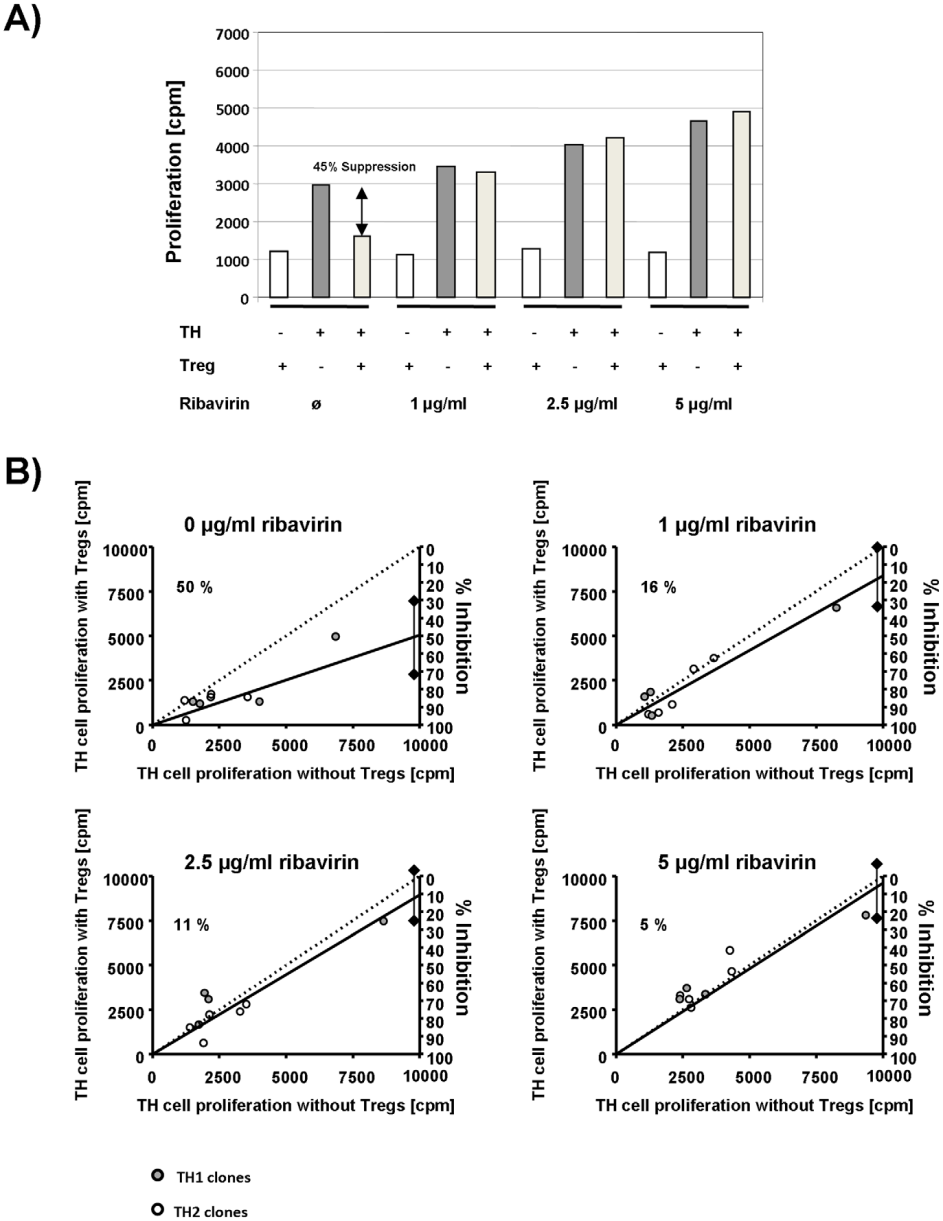
Reversal of Treg-mediated inhibition of T effector cell proliferation by ribavirin. Figure 4A illustrates a representative co-culture experiment of TH1 clone #18 and Treg clone #4 in the presence of varied concentrations of ribavirin (0–5 µg/ml). Figure 4B provides a summary of all co-culture experiments of TH1 and TH2 effector clones with autologous Treg clones in the presence of varied concentrations of ribavirin (0–5 µg/ml). Each plot summarizes the effects for a defined concentration of ribavirin. The x-coordinate of each plot indicates proliferation (cpm = counts per minute) of a T effector clone without Tregs. The coordinate on the left y-axis shows the corresponding proliferation in the presence of autologous Treg clones (1∶1 ratio). Dots below the dotted line illustrate Treg-mediated inhibition of T effector cell proliferation. The straight line represents the linear regression line of all co-culture experiments. Intersection of this line with the right y-axis shows overall percent inhibition and its 95% confidence interval.

Changes of transcription factor mRNA under ribavirin were calculated as follows: Ct (threshold cycle) values were normalized in a first step by subtracting the Ct value of the housekeeping gene ß-actin control (ΔCt = Ct, target – Ct, control) [24]. Expression of gene-specific mRNA upon ribavirin stimulation was calculated by subtracting the normalized median ΔCt of cells in the presence of ribavirin versus cells without ribavirin (ΔΔCt = median ΔCt, ribavirin – median ΔCt, without ribaviri). Changes in transcription factor mRNA expression are given as 2^−ΔΔCt^ ratios [25].

### Assessment of Treg Suppressor Function Against Autologous TH1 and TH2 Reporter Clones in Co-culture Experiments

Inhibitory capacity of Treg clones was analyzed in co-culture assays with stimulated autologous TH1 or TH2 clones. Effector T cells (5×10^4^ cells/well) were stimulated with anti-CD3/anti-CD28. Treg clones were added at 1∶1 ratio. After 5 days, proliferation was measured via [^3^H] thymidine incorporation.

In addition, we performed co-culture experiments of TH1 and TH2 effector clones with Treg clones in the presence of varied concentrations of ribavirin (0, 1, 2.5, 5 µg/ml). To clarify the role of Treg-produced IL-10 for inhibition of TH1 and TH2 cells, we also studied co-cultures using transwells, which disrupt direct cell-cell contacts (tissue culture inserts, 0.2 µm Nanopore® Membrane, Nunc). In addition, we performed blocking experiments using a neutralising antibody to IL-10 (clone 23738, 10 µg/ml; R&D Systems, Germany).

To clarify the relationship of the ribavirin effect to hepatitis C we also performed co-culture assays using freshly isolated Tregs and CD4^+^ TH cells from healthy donors (CD4^+^ CD25^+^ CD127 ^dim/−^ Regulatory T Cell Isolation Kit II, Miltenyi, Germany) at various concentrations of ribavirin (0, 2.5, 5 µg/ml).

### Statistics

Dose response curves were constructed by non-linear curve fit using an uphill and downhill model with variable slopes as appropriate. Differences between experiments were compared by paired t tests and 1-way ANOVA with correction for repeated measurements. To assess over-all inhbition in the co-culture assays, we plotted proliferation of TH cells in the presence of Tregs versus proliferation without Tregs for each concentration of ribavirin (0–5 µg/ml). Then, Treg-mediated inhibition was calculated from the slope obtained by a linear regression analysis.

All calculations and curve fittings were performed using the GraphPad Prism software package 4.0 (GraphPad Prism, San Diego California, USA). P<0.05 was considered as statistically significant.

## Results

With our cloning strategy we obtained 24 CD4^+^ T cell clones from 12 patients with chronic hepatitis C, which - based on their cytokine profiles and proliferative responses - could definitely be classified as Tregs, TH2 cells or TH1 cells, respectively (table 1). Details on the characteristics of the distinct subtypes of T cell clones have been reported previously [19].

To confirm the regulatory function of our 7 Treg clones from chronic hepatitis C, we performed co-culture assays to determine their inhibitory capacity against autologous TH1 and TH2 clones after anti-CD3/anti-CD28 stimulation (figure 1). These co-culture inhibition assays demonstrated that our Treg clones could substantially suppress proliferation of TH1 and TH2 clones (figure 1). Co-culture in semipermeable transwells, which disrupt direct cell-cell contacts but still permit the exchange of soluble factors, indicated that inhibition of TH1 and TH2 clones was mediated by a soluble molecule, which could be identified as IL-10 in blocking experiments with a neutralizing IL-10 antibody (figure 1).

To determine the direct influence of ribavirin on differentially polarized CD4^+^ T cells from chronic hepatitis C, we stimulated our Treg, TH1 and TH2 clones with anti-CD3/anti-CD28 in the presence of variable concentrations of ribavirin and measured their proliferation (figure 2) and cytokine responses (figure 3). Ribavirin did not alter expression of surface markers in Tregs which are considered to be involved Treg-mediated suppression (CTLA-4 ΔMFI: 1.7±3.1%; CD39 ΔMFI: 1.4±2.6%). Furthermore, ribavirin had no effect on the proliferation of Treg clones under normal tissue culture conditions (figure 2A). However, proliferation of both TH2 clones (figures 2B) and TH1 clones (figure 2C) was enhanced in a dose-dependent fashion.

Concerning Treg and TH2 clones, we observed that adding ribavirin to Tregs dose-dependently resulted in reduced IL-10 responses (figure 3A), while its addition to TH2 clones had only minor effects on IL-10 production (figure 3B). On the other hand, addition of ribavirin to TH1 clones, which do not produce IL-10 but IFN-gamma, markedly augmented their cytokine production (figure 3C). However, ribavirin did not significantly alter expression of transcription factors T-bet and GATA-3 in TH1 and TH2 clones (T-bet: 1.34±0.78, GATA-3: 1.55±0.82; 2^−ΔΔCt^ ± SD).

Tregs which do not proliferate under normal culture conditions can be expanded in vitro by adding high amounts of recombinant IL-2. To find out if ribavirin affects proliferation of Tregs during IL-2 induced expansion, we also studied proliferation of anti-CD3/anti-CD28 stimulated Tregs in the presence of 100 IU/ml recombinant IL-2. As it is shown in figure 4, adding ribavirin inhibited IL-2 driven Treg expansion in a dose-dependent fashion.

Ribavirin is frequently used in combination with IFN-alpha. Therefore, we also studied whether the ribavirin-associated effects were detectable in the presence of IFN-alpha. Of note, ribavirin still inhibited IL-10 secretion in Tregs in the presence of 100 IU/ml IFN-alpha (57% inhibition of IL-10 production at 5 µg/ml ribavirin in the presence of 100 IFN-alpha).

To clarify the relationship between HCV infection and effects of ribavirin on Treg functions we also studied Treg clones from individuals with spontaneous recovery from HCV-infection. Of note, adding ribavirin to Treg clones from self-limited HCV infection did not alter IL-10 production (0 µg/ml ribavirin: 176±66; 1 µg/ml: 172±89; 5 µg/ml: 151±82; 10 µg/ml: 143±83, pg/ml IL-10; MW ± SD).

Finally, we performed co-culture inhibition assays in the absence and presense of various ribavirin concentrations. Adding ribavirin to co-culture assays using freshly isolated CD4^+^ CD25^+^ Tregs and autologous CD4^+^ TH cells from healthy donors did not result in abrogation of Treg-mediated suppression (2.5 µg/ml ribavirin: 27±22% inhibition; 5 µg/ml ribavirin: 37±17% inhibition). In contrast, adding ribavirin to co-culture assays of Treg clones from patients with chronic hepatitis C resulted in a dose-dependent reversal of Treg-mediated inhibition of T effector cell proliferation (figure 5).

## Discussion

Ribavirin was initially designed as a direct antiviral agent for the treatment of distinct viral disease, and later on proved to be a pivotal combination partner to enhance efficacy of IFN-based therapy and probably also of direct antiviral agents in chronic hepatitis C [2], [26]. When inhibition of HCV replication could not be confirmed later on, it was proposed that ribavirin might exert effects on host immunity in chronic hepatitis C.

This study used differently polarized CD4^+^ T cell clones from patients with chronic hepatitis C to analyse the effects of ribavirin on T cell functions in vitro. Our experiments revealed directly enhanced proliferation of T effector cells independently from their TH1/TH2 polarization. Furthermore, TH1 cells produce more IFN-gamma, while only little effect on IL-10 production was noted in TH2 cells. Our data largely correspond to similar in vitro studies in mice and human T cells [10], [12]–[14]. Taken together these in vitro findings have been considered to reflect a ribavirin-induced shift in the overall immune balance towards improved antiviral immunity. Nevertheless, ex vivo data remained controversial in this respect [5], [9], [15], but lower production of IL-10 remained the key difference between responders and non-responders to PEG-IFN/ribavirin combination therapy.

Of note, previous studies had been done in preparation of peripheral blood mononuclear cells and thus could not attribute changes in cytokine production to individually polarized T cells. In line with these previous reports our study in CD4^+^ T cell clones confirmed a conspicuous direct effect of ribavirin on TH1 cells, which may be linked to altered expression of interferon regulatory factors [27]–[29]. On the other hand, our data suggest that changes in TH2 functions are unlikely to contribute much to the overall anti-HCV effect of ribavirin, because we observed only slightly reduced production of IL-10 in our TH2 clones.

CD4^+^ Tregs are an alternative source of immunomodulatory cytokines such as IL-10. Thus far, however, frequencies of Tregs in liver and blood have not shown a clear pattern to treatment outcomes [30]–[33]. In two recent studies numbers of Tregs remained unchanged in peripheral blood, whereas exposure to PEG-IFN/ribavirin resulted in increased frequencies of Tregs in the liver [30], [32]. None of the previous studies, however, has studied functions of single Tregs and specifically addressed the potential role of ribavirin.

Here, our in vitro analysis of Treg clones revealed the novel finding that ribavirin markedly reduced production of IL-10 in Tregs, but did not affect their overall low proliferative capacity.

Beyond that, our co-culture assays showed that ribavirin-mediated inhibition of IL-10 production of Treg clones clearly resulted in improved proliferation of T effector cells irrespective from their antigen-specificity and functional polarization.

Our data suggest that ribavirin specifically affects Tregs in patients with chronic hepatitis C, because we observed only negligible effects in Treg clones from patients with self-limited HCV infection and Tregs from healthy controls. Nevertheless we did not check explicitly antigen-specificity of the T cell clones in the current experiments. However, we have shown previously that our Treg clones recognize epitopes on HCV core in an antigen-specific, HLA-DR-restricted fasion [19]. Thus, it is quite likely that the newly discovered action of ribavirin on Tregs holds also true for Tregs in patients with chronic hepatitis C. Moreover, we demonstrated inhibitory activity of ribavirin on Treg function at concentration levels which are typically achieved in vivo during ribavirin treatment (1–5 µg/ml) [21], [22]. Moreover, the ribavirin effect could also be demonstrated at therapeutic concentrations of IFN-alpha (100 IU/ml) suggesting that our in vitro findings might have relevance for the in vivo situation under interferon/ribavirin combination therapy.

Overall, we did not find that ribavirin modulate transcription factors regulating TH1/TH2 cell responses. However, Brenndörfer et al. recently demonstrated that ribavirin can reverse the HCV NS3/4A-mediated impairment of antiviral signalling pathways in vivo [34].

In summary our analysis of T cell clones supports the concept of immune-mediated activity of ribavirin and suggests that this compound affects the T cell balance by two supplementary mechanisms: ribavirin directly enhances proliferation and cytokine production of TH1 cells, and importantly also reverses Treg-mediated inhibition of T effector cells by inhibiting IL-10 release of HCV-specific Tregs. Beyond that, in vivo reduced IL-10 production under ribavirin may also result in down-regulated expression of ICOS, altered costimulatory signalling and increased priming of T cells towards TH1 differentiation [35].

Thus, in chronic hepatitis C ribavirin is likely to shift the functional balance in the immune system between Tregs and T effector cells towards more efficous effector responses, thus strengthening the antiviral activity of PEG-IFN/ribavirin combination therapy.
